# Integrated Sensing and Communication via Orthogonal Time Frequency Space Signaling with Hybrid Message Passing Detection and Fractional Parameter Estimation

**DOI:** 10.3390/s23249874

**Published:** 2023-12-16

**Authors:** Ji Zhang, Leqi Cai, Huanyou Liu

**Affiliations:** 1 China Airborne Missile Academy, Luoyang 471009, China; 2 Intelligent System Science and Technology Innovation Center, Longmen Laboratory, Luoyang 471009, China; 3 School of Mathematics and Statistics, Henan University of Science and Technology, Luoyang 471023, China; 4 Department of Electronics and Electrical Engineering, Southern University of Science and Technology, Shenzhen 518055, China; 12112046@mail.sustech.edu.cn

**Keywords:** OTFS, radar sensing, fractional parameter estimation, hybrid message passing, delay-Doppler (DD) domain

## Abstract

For the orthogonal time frequency space (OTFS) modulation, we generally multiplex symbols on a new type of carrier waveform in the delay-Doppler (DD) domain. These two parameters can be used to infer the range (R) and velocity (V) of the communication user and sensing target; thus, it is natural for the OTFS to be implemented in integrated sensing and communication (ISAC). A framework for ISAC based on OTFS modulation is proposed in this paper, in which the matched filter scheme with fractional parameter estimation is implemented for radar sensing. In addition, the hybrid message passing (MP) detection algorithm is developed for OTFS symbol demodulation. According to the simulation results, fractional DD shifts associated with multiple targets can be accurately obtained through the proposed framework. Meanwhile, the bit error rate under the proposed detector is less than $10^{-4}$ when the signal-to-noise ratio is high enough.

## 1. Introduction

The wireless systems of the future are poised to facilitate wireless communication of superior quality as well as to provide remarkably precise sensing services. The consensus is widespread that sensing capabilities within the forthcoming generation of wireless networks will hold substantially greater significance compared to the networks currently in operation [1,2,3,4].

The majority of scholarly investigations are directed towards orthogonal frequency division multiplexing (OFDM) as a means to achieve the amalgamation of integrated sensing and communication (ISAC) [5,6,7,8,9,10,11,12]. The integration of sensing and communication in the context of ISAC using OTFS signals holds significant promise for addressing challenges in dynamic and multipath-rich environments. ISAC with OTFS signals provides a unified framework where sensing and communication functionalities are seamlessly integrated. This integration allows for joint optimization of resources and enhanced performance in scenarios where both tasks are critical. The DD domain representation in the OTFS provides a powerful way to characterize the environment. In sensing applications, parameters such as target range, velocity, and direction can be directly related to specific regions in the DD domain. OFDM boasts diverse merits, including advantages such as reduced detection intricacy and heightened resilience in radar target detection. Nonetheless, the new requirements for communication and sensing systems pose new challenges for OFDM modulation. For instance, the challenge posed by the increased peak-to-average-power ratio (PAPR) in OFDM systems may hinder power efficiency, particularly concerning carriers at higher frequencies. Additionally, instances of high mobility bring about the deterioration of sub-carrier orthogonality due to the pronounced Doppler effect, thereby potentially leading to considerable degradation in communication performance. Moreover, the channel response in scenarios characterized by substantial mobility exhibits notable fluctuations across distinct coherence regions. In such scenarios, OFDM requires frequent channel estimation to obtain accurate channel state information, leading to a significant increase in signaling overhead. These difficulties serve as a source of inspiration for researchers, propelling them to devise an innovative modulation technique with the capacity to guarantee resilient communication performance in situations characterized by significant mobility.

OFDM and OTFS modulation are different modulation and signal processing techniques designed to address distinct challenges in communication systems. While OFDM is widely used in various current systems, including 4G and 5G cellular networks, OTFS modulation is specifically designed to excel in high-mobility scenarios and challenging channel conditions.

1. Channel Characteristics: OTFS modulation is explicitly designed to handle time-varying channels, making it well suited to high-mobility scenarios. It excels in scenarios with rapid changes in the channel, such as vehicular communication or high-speed rail.
2. Mobility and Doppler Tolerance: OTFS modulation is designed to handle scenarios with high Doppler shifts. Its DD domain representation naturally separates signals, enabling effective mitigation of Doppler effects.
3. Multipath Handling: OTFS modulation excels in multipath environments due to its ability to separate signals in the DD domain, providing superior performance in challenging propagation conditions. OTFS modulation may have an increased computational complexity compared to OFDM.
4. Accuracy in Time-Varying Channels: OTFS modulation excels in accuracy in time-varying channels, offering robust communication and sensing capabilities in high-mobility environments.

The newly unveiled OTFS modulation has garnered considerable attention on account of its prospective capability to enable reliable communication in contexts marked by elevated mobility and multi-path propagation, as pointed out in [13,14,15,16,17].

The OTFS modulation framework is designed to address challenges in communication systems operating in highly dynamic environments with severe multipath and time-varying channel conditions. Under environmental conditions with high interference levels or fluctuating signal strength, the OTFS modulation framework can also demonstrate its advantages. For example:

1. Multipath Fading and Time Variability
  (a) Performance Improvement: OTFS modulation is particularly well suited to environments with severe multipath fading. By transforming the received signals from the time-delay domain to the DD domain, OTFS modulation can effectively separate multipath components. This transformation enhances the system’s ability to combat fading and improves communication performance.
  (b) Resilience to Time Variability: OTFS modulation is designed to handle time-varying channels, making it robust in scenarios where the channel conditions change rapidly. This is crucial in dynamic environments, such as those encountered in vehicular communication or mobile communication, where the channel characteristics can vary over short time intervals.
2. Interference Rejection
  (a) Spatial Separation: The DD domain representation in OTFSs provides a form of spatial separation for different signals. This can aid in the rejection of interference, as signals from different directions in the DD domain can be more easily distinguished.
  (b) Improved Detection in Noisy Environments: The ability of OTFS modulation to focus on distinct DD signatures allows for improved detection in the presence of interference and noise. The framework’s capacity to isolate signals in the DD domain contributes to enhanced interference rejection capabilities.

The OTFS modulation framework shows promise in addressing challenges posed by varying environmental conditions, including interference and fluctuating signal strengths. Its unique approach of focusing on the DD domain provides advantages in multipath fading scenarios and dynamic channel conditions, making it a candidate for applications requiring reliable communication in challenging environments. In contrast to conventional approaches that operate in the time-frequency (TF) domain, the OTFS modulation strategy conveys information symbols (ISs) across the two-dimensional (2D) DD domain, as explained in [14,18,19,20,21,22]. In this domain, wireless channel paths with distinct DD shifts represent resolvable paths. Significant benefits are associated with employing the DD domain for channel characterization. In communication scenarios featuring pronounced mobility, the DD domain channel typically exhibits sparsity and quasi-static behavior in contrast to the highly dynamic properties in the TF domain, as emphasized in references [23,24,25,26]. Additionally, the OTFS boasts a reduced PAPR [25,27,28,29,30], simplifying implementation through more efficient power amplifiers, unlike its OFDM counterpart. In traditional OFDM systems, a high PAPR requires power amplifiers to operate with a larger dynamic range, leading to increased power requirements and impacting the overall energy efficiency of the system. OTFS modulation utilizes a unique signal representation in the DD domain, which may impact the PAPR characteristics differently compared to traditional OFDM. The energy efficiency of the OTFS modulation framework may be influenced by the computational complexity of its signal processing algorithms. Adaptive techniques for power control and modulation parameters may be employed to manage power levels dynamically, contributing to energy efficiency. Notably, the DD shifts, which correspondingly infer the velocity and range of targets, are pivotal, making OTFS modulation a logical selection for achieving ISAR (inverse synthetic aperture radar) objectives.

Recent scholarly endeavors have examined radar sensing using the OTFS framework, as evidenced in [31,32]. A maximum likelihood (ML) algorithm for estimating R and V in the OTFS is introduced by the authors in [31]. The results demonstrate that the OTFS signal has superior communication effectiveness compared to OFDM while preserving the radar estimation performance limit. Furthermore, Ref. [32] proposed a matched-filter (MF) algorithm to estimate the R and V parameters, leveraging the structure of the OTFS effective channel (EC). The findings indicate that OTFS-based speed estimations of targets outperform those of OFDM.

Nevertheless, the potential of employing OTFS modulation for radar sensing remains largely untapped. To illustrate, the complexity of the algorithm introduced in [31] increases almost threefold with respect to the size of the OTFS frame, indicating a relatively elevated computational load. Conversely, the investigation carried out in [32] solely accounts for integer-valued DD shifts. Nonetheless, this approach might prove impractical in real-world wireless networks, where temporal resources are constrained, leading to inadequate Doppler resolution. As a result, the inclusion of fractional Doppler components becomes inevitable. Furthermore, achieving precise sensing performance mandates an exploration of radar sensing with limited frequency resources, even though integer delay suffices for communication design in most cases. Consequently, considering both fractional DD indices becomes imperative for effective radar sensing. These concerns collectively drive the motivation to develop an approach that facilitates radar sensing through OTFS signaling, accommodating fractional DD shifts.

The practical implementation of OTFS modulation is facilitated by its compatibility with conventional OFDM and pulse-shaped OFDM transceiver structures. This is achieved through the straightforward addition of pre-processing and post-processing blocks. The choice of shaping pulses in a communication or sensing system can significantly impact the overall performance and the spectral efficiency, especially in the context of sensing where accurate channel estimation and signal recovery are crucial. Shaping pulses are waveforms that are used to shape the transmitted signal and, when appropriately chosen, can enhance various aspects of the sensing performance. By using pulses with well-designed spectral characteristics, it is possible to optimize the use of available bandwidths. The choice of shaping pulses influences the duration and bandwidth of the transmitted signal. Depending on the sensing requirements and the characteristics of the environment, it may be desirable to use pulses with specific durations and bandwidths.

In addition, to achieve full diversity in TF (time frequency) considerations, effective detection methods are essential. Implementing the maximum a posteriori algorithm (MAP) for each symbol, as detailed in reference [33,34,35,36,37,38], represents an optimal approach for data detection within interference channels. The MAP algorithm can be represented by an intuitive cyclic factor graph. However, it is crucial to note that the MAP algorithm becomes computationally infeasible since the number of computation-interfering elements grows exponentially. This exponential growth renders it impractical for scenarios involving multiple signal paths, which requires a low-complexity detector.

In an effort to mitigate this computational burden, numerous approximate methods have been developed. For instance, in reference [39], an alternative MAP algorithm is introduced, which leverages Gaussian approximation (GA) to reduce the complexity of these procedures. Reference [9] explores the use of detectors founded on approximate message passing (AMP). Furthermore, reference [40] introduces a unitary approximate message passing detector, which has the same detection performance as the minimal mean square error (MMSE). Lastly, reference [41] presents a detector based on variational Bayes (VB), which has significantly lower computational demands while ensuring convergence with significantly lower computational demands.

This paper introduces a method for estimating fractional DD method. First, the radar receiver captures the echoes reflected by targets in the DD domain and subsequently carries out 2D matched filtering involving the DD domain symbols between the receiver and transmitter. With the correlation matrix in hand, we deploy a difference-based technique that entails computing the discrepancy between the indices corresponding to the highest and second highest magnitudes in the correlated matrix. This calculation allows us to determine the fractional DD indices. For the OTFS symbol detection part, a novel hybrid MP detection algorithm has been introduced. When fractional Doppler effects are present, the channel responses are dispersed across the entire Doppler spectrum. This dispersion leads to more interfering symbols and a high computational complexity. However, only a subset of path gains corresponding to a specific Doppler frequency shifts rather than the whole dispersion, which will significantly affect the transmission of the OTFS signal [42,43,44,45,46]; therefore, a selective approach is employed. Specifically, data detection is implemented based on the paths with the highest gains, which are named main paths, associated with the dispersed Doppler indices. We employed a non-approximated MP algorithm for the primary propagation paths, whereas a GA-based MP algorithm was used for the secondary propagation paths. Through our simulation results, we illustrate its effectiveness regarding accuracy.

## 2. System Model

In this section, we first introduce the fundamental principles underlying OTFS modulation. Subsequently, we expound upon the input–output correction of OTFS symbols within the DD domain [47,48,49,50,51,52].

### 2.1. Introduction of OTFS Modulation

We utilize the symbols $N_{T}\in N$ and $M_{S}\in N$ to signify the quantity of time slots and subcarriers allocated to each OTFS frame, respectively, with $N_{T}$ belonging to the set of positive integers. The discrete DD plane can be succinctly depicted as follows:

(1) $$\Gamma =\left\{\left(\frac{k}{N_{T}T},\frac{l}{M_{S}\Delta f}|k\in \left[0,N_{T}-1\right],l\in \left[0,M_{S}-1\right]\right)\right\}.$$

The duration of a single time slot can be represented by *T*, and the separation between subcarriers is denoted as Δf. Consequently, the entire duration of an OTFS frame is given by $N_{T}T$, while the occupied bandwidth of the system is $M_{S}\Delta f$. In terms of resolution, we attain a delay resolution of $\frac{1}{M_{S}\Delta f}$ and a Doppler shift resolution of $\frac{1}{N_{T}T}$.

The cross-ambiguity function (CAF) between the pulse-shaping filters (PSFs) at the transmitter $g_{tx}\left(t\right)$ and the receiver $g_{rx}$ can be expressed as follows:

(2) $$A_{g_{rx}g_{tx}}\left(t,f\right)\overset{\Delta }{=}\int_{t}g_{tx}\left(t^{\prime }\right)g_{rx}^{*}\left(t^{\prime }-t\right)e^{j2\pi \nu (t^{\prime }-t)}dt^{\prime }.$$

To ensure clarity in our discourse, we focus our analysis on situations in which both the transmitter and receiver operating within the OTFS framework are endowed with ideal PSFs. As elucidated in [53], these filters exhibit bi-orthogonal characteristics, which is delineated as follows:

(3) $$\begin{array}{cc} & A_{g_{rx}g_{tx}}{\left(t,f\right)|}_{t=\left(n-n^{\prime }\right)T-\frac{l_{\tau }}{M_{S}\Delta f},\left(m-m^{\prime }\right)\Delta f-\frac{k_{\nu }}{N_{T}T}}\;\\ \; & =\delta \left[n\right]\delta \left[m\right]q_{\tau_{max}}\left(t-nT\right)q_{\nu_{max}}\left(f-m\Delta f\right), \end{array}$$

where

$$q_{a}\left(t\right)=\left\{\begin{array}{c} 1\;-a<x<a\\ 0\;otherwise. \end{array}\right.$$

While the OTFS framework presents promising capabilities for addressing challenges in wireless communication and sensing systems, there are several practical challenges that need to be considered during implementation in real-world scenarios, including:

1. Computational Complexity: The OTFS framework involves complex mathematical transformations, including the conversion from the time-delay domain to the DD domain. These operations can be computationally intensive, especially in real-time applications or resource-constrained environments.
2. Channel Estimation and Tracking: Accurate channel estimation and tracking are crucial for the success of the OTFS framework. In dynamic environments with rapidly changing channel conditions, developing robust algorithms for real-time channel estimation and tracking becomes a challenge.
3. Synchronization: Achieving precise synchronization is essential for accurate transformation between domains in the OTFS framework. Synchronization challenges may arise in scenarios with high mobility, multipath propagation, and varying signal delays.
4. Adaptability to Non-Ideal Conditions: Real-world environments often introduce non-ideal conditions such as frequency-selective fading, non-line-of-sight (NLOS) scenarios, and interference. The OTFS framework needs to be adaptable to such conditions, requiring advanced signal processing techniques and algorithms.

While the OTFS framework shows promise, its successful implementation in real-world wireless communication and sensing systems requires careful consideration of these practical challenges.

### 2.2. DD Domain Input–Output Relationship in OTFS Modulation

We will now delve into a comprehensive elucidation of the OTFS transmission process. To begin with, a sequence of information bits is subjected to a mapping procedure, resulting in a symbol set situated within the DD domain, as described in previous references [54,55,56,57,58]. This symbol set is formally denoted as $\{X_{\mathrm{DD}}\left[k,l\right]|k=0,\ldots ,N_{T}-1,$ $l=0,\ldots ,M_{S}-1\}$. It is essential to underscore that both the indices k and l correspond to the DD references, respectively. Furthermore, it is worth noting that $X_{\mathrm{DD}}$ belongs to the modulation alphabet set A, defined as $A=\left\{a_{i}\in C|i=1,2,\ldots ,|A|\right\}$, encompassing a range of modulation schemes, including quadrature amplitude modulation (QAM). Subsequently, the OTFS symbols undergo a transformation using the inverse symplectic (IS) FFT. This transformation process will be further elaborated upon in the subsequent discussion.

(4) $$X_{\mathrm{TF}}\left[n,m\right]=\sum_{k=0}^{N_{T}-1}\sum_{l=0}^{M_{S}-1}X_{\mathrm{DD}}\left[k,l\right]e^{j2\pi \left(\frac{nk}{N_{T}}-\frac{ml}{M_{S}}\right)},$$

for $n=0,\ldots ,N_{T}-1$ and $m=0,\ldots ,M_{S}-1$.

In the next step, the modulator, which functions in the TF domain, processes the symbols $X_{\mathrm{TF}}\left[n,m\right]$, transforming them into a continuous-time waveform represented as s(t). This conversion is realized by harnessing the transmitter’s pulse-shaping function (PSF) $g_{tx}\left(t\right)$, making use of the Heisenberg transform technique as detailed in reference [59].

(5) $$s\left(t\right)=\sum_{n=0}^{N_{T}-1}\sum_{m=0}^{M_{S}-1}X_{\mathrm{TF}}\left[n,m\right]g\left(t-nT\right)e^{j2\pi m\Delta f(t-nT)}.$$

Following its propagation through a time-varying channel, as elucidated in reference [60], the signal, initially denoted as s(t), reaches the receiver. So, we can obtain the received signal, designated as r(t):

(6) $$r\left(t\right)=\int \int h\left(\tau ,\nu \right)s\left(t-\tau \right)e^{j2\pi \nu (t-\tau )}d\tau d\nu +w\left(t\right).$$

Within this equation, the term w(t) signifies the existence of additive white Gaussian noise (AWGN), represented as $N_{0}$. On the other hand, h(τ,ν)∈C denotes a complex baseband channel impulse response, where τ and ν represent the channel’s reaction to an impulse with DD shift (τ and ν, i.e.). The equation is

(7) $$h\left(\tau ,\nu \right)=\sum_{i=1}^{P}h_{i}\delta \left(\tau -\tau_{i}\right)\delta \left(\nu -\nu_{i}\right).$$

In this context, our notation includes P∈Z to represent the number of targets present. We further employ $h_{i}$, $\tau_{i}$, and $\nu_{i}$ to signify the attributes of the ith target, specifically the reflection DD shift and channel coefficient, respectively. Additionally, the velocity and range of the *i*th target are denoted by $R_{i}$ and $v_{i}$. Within this framework, we can express the corresponding Doppler frequency $\nu_{i}\in R$ and delay $\tau_{i}\in R$ in a mathematical formulation.

(8) $$\tau_{i}=\frac{2R_{i}}{c}=\frac{l_{\tau_{i}}}{M_{S}\Delta f},\nu_{i}=\frac{2f_{c}v_{i}}{c}=\frac{k_{\nu_{i}}}{N_{T}T}.$$

Subsequently, we establish a notation where $f_{c}$ denotes the carrier frequency and *c* represents the speed of light. Furthermore, we introduce the utilization of $l_{\tau_{i}}\in R$ and $k_{\nu_{i}}\in R$ as the indices characterizing Doppler and delay properties for the *i*th target. In the context of this study, we expand our analysis to encompass fractional elements of Doppler and delay. These indices, specifically $l_{\tau_{i}}=l_{i}+\iota_{i}$ and $k_{\nu_{i}}=k_{i}+\kappa_{i}$, incorporate the following elements: $l_{i}\in Z$ and $k_{i}\in Z$, while $\iota_{i}\in R$ and $\kappa_{i}\in R$ signify the real numbers representing the fractional aspects.

Upon reception at the receiver, the incoming signal, denoted as r(t), is subjected to a transformation to the TF domain. This transformation is executed through the application of the Wigner transform, as elaborated in reference [59]. It is paramount to underscore that this Wigner transform can equivalently be construed as the CAF, denoted as $A_{g_{rx},r}\left(t,f\right)$. Furthermore, it may be formally defined as:

(9) $$\begin{array}{cc} \; & Y_{\mathrm{TF}}\left(t,f\right)=A_{g_{rx},r}\left(t,f\right)\\ \; & \overset{\Delta }{=}\int g_{rx}^{*}\left(t^{\prime }-t\right)r\left(t^{\prime }\right)e^{-j2\pi f(t^{\prime }-t)}dt^{\prime }. \end{array}$$

Upon performing the sampling of Y(t,f), we derive the discrete output in the following manner:

(10) $$Y_{\mathrm{TF}}\left[n,m\right]=Y_{\mathrm{TF}}{\left(t,f\right)|}_{t=nT,f=m\Delta f},$$

for $m=0,\ldots M_{S}-1$ and $n=0,\ldots ,N_{T}-1$. Consequently, through the application of the SFFT to $Y_{\mathrm{TF}}\left[n,m\right]$, we obtain the discrete output symbols $Y_{\mathrm{DD}}\left[k,l\right]$ in the DD domain.

(11) $$Y_{\mathrm{DD}}\left[k,l\right]=\frac{1}{\sqrt[{}]{N_{T}M_{S}}}\sum_{n=0}^{N_{T}-1}\sum_{m=0}^{M_{S}-1}Y_{\mathrm{TF}}\left[n,m\right]e^{-j2\pi \left(\frac{nk}{N_{T}}-\frac{ml}{M_{S}}\right)}.$$

In cases where an ideal PSF is employed, as elaborated in reference [61], the IO relationship between the OTFS symbols can be described as:

(12) $$\begin{array}{cc} Y_{\mathrm{DD}}\left[k,l\right]=\sum_{n=0}^{N_{T}-1}\sum_{m=0}^{M_{S}-1} & X_{\mathrm{DD}}\left[n,m\right]h_{\omega }\left[k-n,l-m\right]\\ \; & +Z_{\mathrm{DD}}\left[k,l\right]. \end{array}$$

Here, $Z_{\mathrm{DD}}$ adheres to a complex Gaussian distribution characterized by a mean of zero and a covariance of $\sigma^{2}I$, representing the the DD domain effective noise. Additionally, $h_{\omega }\left[k,l\right]$ is the EC in the DD domain, acquired via the following means:

(13) $$h_{\omega }\left[k,l\right]=\sum_{i=1}^{P}h_{i}\omega \left(k-k_{\nu_{i}},l-l_{\tau_{i}}\right)e^{-j2\pi \nu_{i}\tau_{i}}.$$

In this context, the function $\omega (k-k_{\nu_{i}},l-l_{\tau_{i}})$ serves as the sampling function. The parameters $\nu_{i}$ and $\tau_{i}$ are defined in accordance with Equation (8). When both the TF transmitter and receiver opt to employ a rectangular window, as extensively elucidated in reference [53], the sampling function takes on a more streamlined and simplified form, as follows:

(14) $$\begin{array}{cc} \; & \omega \left(k-k_{\nu_{i}},l-l_{\tau_{i}}\right)=G\left(k-k_{\nu_{i}}\right)F\left(l-l_{\tau_{i}}\right),\\ \; & G\left(k-k_{\nu_{i}}\right)\overset{\Delta }{=}\frac{1}{N_{T}}\sum_{k^{\prime }=0}^{N_{T}-1}e^{-j2\pi \frac{\left(k-k_{\nu_{i}}\right)k^{\prime }}{N_{T}}},\\ \; & F\left(l-l_{\tau_{i}}\right)\overset{\Delta }{=}\frac{1}{M_{S}}\sum_{l^{\prime }=0}^{M_{S}-1}e^{j2\pi \frac{\left(l-l_{\tau_{i}}\right)l^{\prime }}{M_{S}}}. \end{array}$$

As expounded in reference [61], the non-zero components of the EC in the DD domain exhibit uniform localization patterns when both the rectangular and ideal PSFs are utilized. The primary distinction lies in the introduction of an additional phase offset. Consequently, the algorithms delineated in this study can be readily and seamlessly adapted to suit scenarios where a rectangular PSF is employed.

## 3. OTFS-Based Radar Sensing

In this section, we introduce a target detection method and the 2D MF-based technique to effectively estimate the parameters.

### 3.1. Two-Dimensional Matched-Filter Operation

In this subsection, our objective is to provide a detailed explanation of the 2D MF operation for integer parameter estimation. When we initially examine the representation of the received OTFS symbol matrix in the DD domain, the direct identification of target elements within $Y_{\mathrm{DD}}$ (received data in the DD domain) becomes challenging due to the presence of IS. These symbols are influenced by the time-varying channel, which leads to the emergence of overlapping responses in the received DD domain signals. These responses arise from both the channel characteristics and the superimposed IS. In this context, we draw inspiration from pulse compression radar sensing and implement a 2D MF-based estimator. This estimator is likened to performing simultaneous pulse compression in both the DD dimensions, which, in turn, allows us to effectively capture the DD parameters.

To represent the matrix associated with the 2D MF as V, the diverse DD indices of the MF coefficient are denoted as:

(15) $$V\left[k,l\right]=\sum_{n=0}^{N-1}\sum_{m=0}^{M_{S}-1}Y_{\mathrm{DD}}^{*}\left[n,m\right]X_{\mathrm{DD}}\left[{\left[n-k\right]}_{N_{T}},{\left[m-l\right]}_{M_{S}}\right].$$

**Figure 1:**
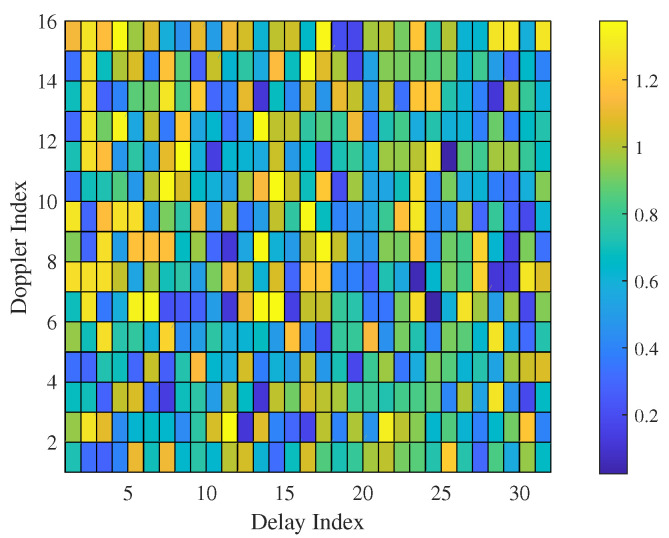
The illustration of the received matrix under P=3 targets.

To provide a more lucid explanation of the proposed approach, we have devised a simplified radar sensing example using OTFS principles. In this illustrative scenario, we assume the existence of five targets, denoted as P=5. The symbols were randomly generated from a four-QAM scheme. We set the parameters $M_{S}$ to 32 and $N_{T}$ to 16, with specific DD indices assigned to each target, represented as {4,9,3} and {7,1,4}, respectively. The resulting symbol matrix in the DD domain, under this particular configuration, is visually represented in Figure 1. As illustrated, the matrix $Y_{\mathrm{DD}}$ exhibits a dense population, primarily attributable to the overlapping responses originating from the DD domain symbols. The task of target identification within this matrix is inherently intricate. However, through the application of a 2D MF computation, the targets become more localized. This process can be likened to a specialized form of pulse compression in the DD domain, analogous to its role in radar sensing. Ultimately, this improves the retrieval of responses in the DD domain.

### 3.2. GLRT-Based Target Detection

As we encounter the challenge of not knowing the precise number of targets when working with the 2D MF matrix, we have devised a step-by-step algorithm to identify them systematically. Once a target is located, we can simultaneously estimate its crucial characteristics. We continue this process until no more targets can be found.

Let us rewrite Equation (15) in a different form using matrices before we get into the details of the target detection algorithm. This will simplify the detection process and enhance comprehension. We achieve this by substituting Equations (13) and (12) into Equation (15) and performing straightforward calculations.

(16) $$v=\sum_{i=1}^{P}h_{i}^{*}e^{j2\pi \frac{l_{\tau_{i}}k_{\nu_{i}}}{M_{S}N_{T}}}\tilde{X}W_{\left(\tau_{i},\nu_{i}\right)}^{H}x^{*}+{\tilde{X}}^{T}z^{*},$$

in which the vector $x^{*}$ has an *i*-th entry in the matrix X at row $k_{1}$ and column $l_{1}$, and $\tilde{X}$ is a rearranged matrix of transmitted symbols, which are circulant shifts of the symbols in X. The specific element of the matrix X located at row $k_{1}$ and column $l_{1}$ is represented as follows:

(17) $$\tilde{X}\left[i,j\right]=x\left[{\left[k_{1}-k_{2}\right]}_{N_{T}},{\left[l_{1}-l_{2}\right]}_{M_{S}}\right].$$

In this context, where we define $i=k_{1}M_{S}+l_{1}$ and $j=k_{2}M_{S}+l_{2}$, $k_{\left(\cdot \right)}\in \left[0,N_{T}-1\right]$ and $l_{\left(\cdot \right)}\in \left[0,M_{S}-1\right]$ represent row and column indices of the entry in X. The element at position $\left(kM_{S}+l\right)$ in the EC vector h corresponds to the entry in the EC matrix $h_{\omega }$ at row *k* and column *l*, and the EC sampling function is represented in matrix form, i.e., $W_{\left(\tau_{i},\nu_{i}\right)}^{H}$. The element in the matrix W at $i=k_{1}M_{S}+l_{1}$ and $j=k_{2}M_{S}+l_{2}$ is defined as follows:

(18) $$\tilde{W}\left[i,j\right]=\omega \left(k_{2}-k_{1}-k_{\nu_{i}},l_{2}-l_{1}-l_{\tau_{i}}\right),$$

where ω(ν,τ) is precisely defined as outlined in Equation (14).

We conduct a binary hypothesis test to obtain the number of targets. In this test, $H_{0}$ means that the *i*th target is not present, and $H_{1}$ means that the *i*th target is present. The observations can be described as follows:

(19) $$v=\left\{\begin{array}{ccc} \; & \tilde{X}z^{*} & ,\;\mathrm{under}\;H_{0}\\ \; & h^{*}e^{j2\pi \frac{l_{\tau }k_{\nu }}{M_{S}N_{T}}}\tilde{X}W_{\left(\tau ,\nu \right)}^{H}x^{*} & \\ \; & +\tilde{X}z^{*} & ,\;\mathrm{under}\;H_{1} \end{array}\right..$$

To address Equation (19), we consider *h*, τ and ν as deterministic but unknown variables, and use the generalized likelihood ratio test (GLRT) [62]. The inspection is as follows:

(20) $$\Lambda \left(v\right)=\max_{h,\tau ,\nu }\frac{p(v|H_{1};h,\tau ,\nu )}{p(v|H_{0})}\overset{H_{1}}{\underset{H_{0}}{≷}}\eta .$$

Here, where η serves as the threshold, we make the assumption that z adheres to a complex Gaussian distribution characterized by parameters $\mathrm{CN}(0,\sigma^{2})$. Consequently, we can signify that the product ${\tilde{X}}^{T}z^{*}$ also conforms to a complex Gaussian distribution, with parameters $\mathrm{CN}(0,\frac{\sigma^{2}}{M_{S}N_{T}})$. Furthermore, by introducing the term $\tilde{h}=h^{*}e^{j2\pi \frac{l_{\tau_{i}}k_{\nu_{i}}}{M_{S}N_{T}}}$, the GLRT takes the following expression:

(21) $$\Lambda \left(v\right)=\frac{e^{-\frac{1}{\sigma^{2}}\min_{h,\tau ,\nu }{\left\|v-\tilde{h}\tilde{X}W_{\left(\tau ,\nu \right)}^{H}x^{*}\right\|}^{2}}}{e^{-\frac{1}{\sigma^{2}}{\left\|v\right\|}^{2}}}\overset{H_{1}}{\underset{H_{0}}{≷}}\tilde{\eta }.$$

When τ and ν are constant, the value of $\tilde{h}$ in Equation (21) is as follows:

(22) $$\tilde{h}\approx \frac{x^{T}W{\tilde{X}}^{H}v}{{\left\|x\right\|}^{2}}.$$

This approximation arises from the matrix $\frac{{\tilde{X}}^{T}{\tilde{X}}^{*}}{M_{S}N_{T}}$ exhibiting an approximate identity property. The approximately equal sign converges to an equal sign as $M_{S}N_{T}$ approaches infinity. For a more comprehensive proof, we refer interested readers to the detailed explanation provided in reference [32]. By adopting the procedure outlined in reference [32] with a phase offset of zero, we establish the approximate identity property of $\frac{{\tilde{X}}^{T}{\tilde{X}}^{*}}{M_{S}N_{T}}$. Considering the parameter $\tilde{h}$, we can formulate the GLRT:

(23) $$\max_{\tau ,\nu }\frac{|x^{T}W\left(\tau ,\nu \right){\tilde{X}}^{H}v{|}^{2}}{\sigma^{2}{\left\|x\right\|}^{2}}\overset{H_{1}}{\underset{H_{0}}{≷}}\tilde{\eta }.$$

Within this framework, the cell-averaging constant false alarm rate (CA-CFAR) technique is employed to ascertain an adaptive threshold, $\tilde{\eta }=\log\left(\eta \right)$. The methodology for calculating $\tilde{\eta }\left(\nu ,\tau \right)$ is elucidated in the reference authored by Raviteja et al. (2018) [61]. Targets are discerned at locations where the peak magnitudes surpass this specified threshold, with the resulting DD parameters being considered as the attributes linked to these identified targets.

### 3.3. Fractional Parameter Estimation

Upon examining the peaks within the DD plane denoted as Γ, we are limited to discerning only the integer components of the DD indices [63,64,65,66]. This constraint implies that the fractional segments of these respective indices remain undisclosed, thereby diminishing the precision of the sensing outcomes, as elaborated upon in references [67,68,69,70]. To rectify these concerns, this subsection will present an uncomplicated technique for estimating the fractional parameters.

**Figure 2:**
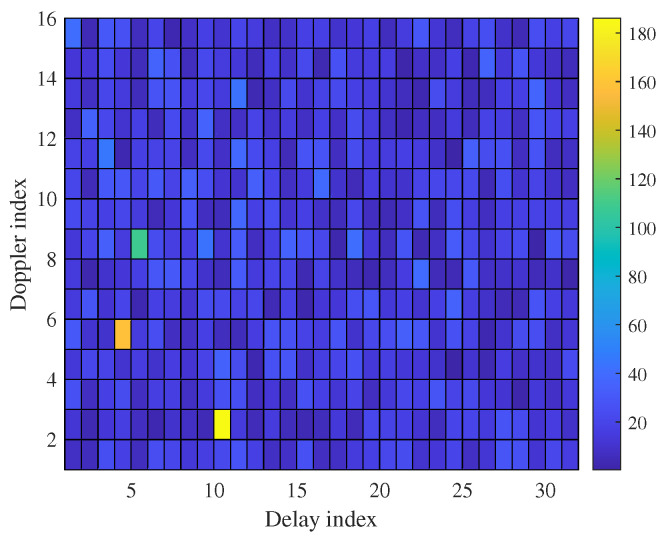
An illustration of the corrected matrix under P=3 targets.

Upon a closer examination of the 2D MF matrix illustrated in Figure 2, it becomes evident that targets with fractional indices exhibit a phenomenon known as power leakage (PL) into neighboring DD bins. A thorough exploration of the origins of this PL resulting from fractional indices is available in [53,71]. Building upon this insight, we introduce a distinctive method for estimating these fractional indices differentially, as detailed below.

To calculate the fractional component of the Doppler index of the *i*-th target, we first assume that the delay or Doppler shift is different for each target. Following the 2D MF operation, the row indices associated with the highest and second highest magnitudes within the $l_{i}$-th column of the DD matrix, denoted as $k_{1}^{\prime }$ and $k_{2}^{\prime }$, are:

(24) $$\begin{array}{cc} k_{1}^{\prime } & =\underset{k\in \left\{\left\lceil -N_{T}/2\right\rceil ,\ldots ,\left\lceil N_{T}/2\right\rceil -1\right\}}{arg max}\left|V\left[k,l_{i}\right]\right|,\\ k_{2}^{\prime } & =\underset{k\in \left\{\left\lceil -N_{T}/2\right\rceil ,\ldots ,\left\lceil N_{T}/2\right\rceil -1\right\}\setminus \left\{k_{\nu_{1}}^{\prime }\right\}}{arg max}\left|V\left[k,l_{i}\right]\right|. \end{array}$$

Upon establishing the values of $k_{1}^{\prime }$ and $k_{2}^{\prime }$, a significant observation comes to light. In noise-free scenarios, the genuine Doppler index, represented as $k_{i}+\kappa_{\nu_{i}}$ and linked to the *i*-th target, logically falls within the interval delineated by $k_{\nu_{1}}^{\prime }$ and $k_{\nu_{2}}^{\prime }$. It is worth emphasizing that $k_{i}$ coincides with $k_{\nu_{1}}^{\prime }$, and the discrepancy between $k_{\nu_{2}}^{\prime }$ and $k_{\nu_{1}}^{\prime }$ precisely equals 1. Moreover, the proportion between the magnitudes of the MF coefficients associated with Doppler indices $k_{\nu_{1}}^{\prime }$ and $k_{\nu_{2}}^{\prime }$ at the same delay index is reasonably estimated in the following manner:

(25) $$\frac{\left|V[k_{\nu_{1}}^{\prime },l_{i}]\right|}{\left|V[k_{\nu_{2}}^{\prime },l_{i}]\right|}\approx \frac{|k_{\nu_{2}}^{\prime }-k_{\nu_{1}}^{\prime }-\kappa_{\nu_{i}}|}{|-\kappa_{\nu_{i}}|}.$$

The error of the approximation above is $O\left(\frac{1}{M_{S}N_{T}}\right)$.

As a result, we can calculate the fractional Doppler value as:

(26) $$\kappa_{\nu_{i}}=\frac{\left(k_{\nu_{2}}^{\prime }-k_{\nu_{1}}^{\prime }\right)\left|V\left[k_{\nu_{2}}^{\prime },l_{i}\right]\right|}{|V\left[k_{\nu_{1}}^{\prime },l_{i}\right]|+|V\left[k_{\nu_{2}}^{\prime },l_{i}\right]|}.$$

Likewise, employing the same approach described above for fractional delay taps, we obtain:

(27) $$\iota_{\tau_{i}}=\frac{\left(l_{\tau_{2}}^{\prime }-l_{\tau_{1}}^{\prime }\right)\left|V\left[k_{i},l_{\tau_{2}}^{\prime }\right]\right|}{\left|V[k_{i},l_{\tau_{1}}^{\prime }]|+|V[k_{i},l_{\tau_{2}}^{\prime }]\right|}.$$

## 4. Hybrid Message Passing Detector

We will formulate the probability model for the proposed symbol detection algorithm in this section. Subsequently, we provide an in-depth explanation of the hybrid MP algorithm.

The DD domain input–output relationship takes on the vectorized form:

(28) $$\begin{array}{c} y=Hx+w. \end{array}$$

In the context at hand, we have several key variables. Firstly, the received signal vector, denoted as y, belongs to the complex vector space $C^{M_{S}N_{T}\times 1}$. Secondly, the EC matrix, denoted as H (which is considered as known at the receiver when performing the symbol detection), belongs to the complex matrix space $C^{M_{S}N_{T}\times M_{S}N_{T}}$. It is noteworthy that H is sparse and characterized by its elements H[d,c]. Moving on, the information vector x is an element of the complex vector space $C^{M_{S}N_{T}\times 1}$, with each component x[c] taking values from the set A and satisfying $1\leq c\leq M_{S}N_{T}$. Finally, we introduce the additive white Gaussian noise vector w, which is an element of the complex vector space $C^{M_{S}N_{T}\times 1}$. To elaborate further, the structure of H is expressed as follows:

(29) $$H=\sum_{i=0}^{P-1}\sum_{j=-N_{i}}^{N_{i}}I_{N}\left(-{\left[j-k_{\nu_{i}}\right]}_{N}\right)⊗\left[I_{M}\left(l_{\tau_{i}}\right)\times \frac{1}{N}\left(\frac{\left.e^{-j2\pi \left(-j-\kappa_{\nu_{i}}\right.}\right)-1}{e^{-j\frac{2\pi }{N}\left(-j-\kappa_{\nu_{i}}\right)}-1}\right)h_{i}e^{-j2\pi \nu_{i}\tau_{i}}\right].$$

In our framework, we have two circular shifting matrices denoted as $I_{N}$ and $I_{M}$. $R_{d}$ and $C_{c}$ are defined as the sets whose the *d*-th row and *c*-th column are non-zero elements in the EC H, respectively. By examining the composition of the channel matrix, it becomes evident that the cardinality of both sets denoted as $|R_{d}|$ and $|C_{c}|$ equals a constant value, which we denote as *S*.

The transmitted signals are estimated by the MAP rule. Referring to Equation (10), this estimation procedure can be expressed as:

(30) $$\begin{array}{c} \hat{x}=\underset{x\in A^{N_{T}M_{S}}}{\arg\max}P\left(x|y,H\right). \end{array}$$

Consider that each of the transmitted symbols in the vector x is assumed to be independently drawn from the constellation set A with equal probability, which is equivalent to a uniform prior for x. Furthermore, given the approximate independence of the components of vector y, we can approximate the a posteriori probability, denoted as $P(x\left[c\right]=a_{j}|y,H)$, as follows:

(31) $$\begin{array}{cc} P\left(x\left[c\right]=a_{j}|y,H\right) & =\frac{1}{Q}P\left(y|x\left[c\right]=a_{j},H\right)\approx \prod_{d\in C_{c}}P\left(|y\left[d\right]|x\left[c\right]=a_{j},H\right). \end{array}$$

The elements $\hat{x}\left[c\right]$ and x[c], which belong to the vectors $\hat{x}$ and x, respectively, satisfy the condition $1\leq c\leq M_{S}N_{T}$.

**Figure 3:**
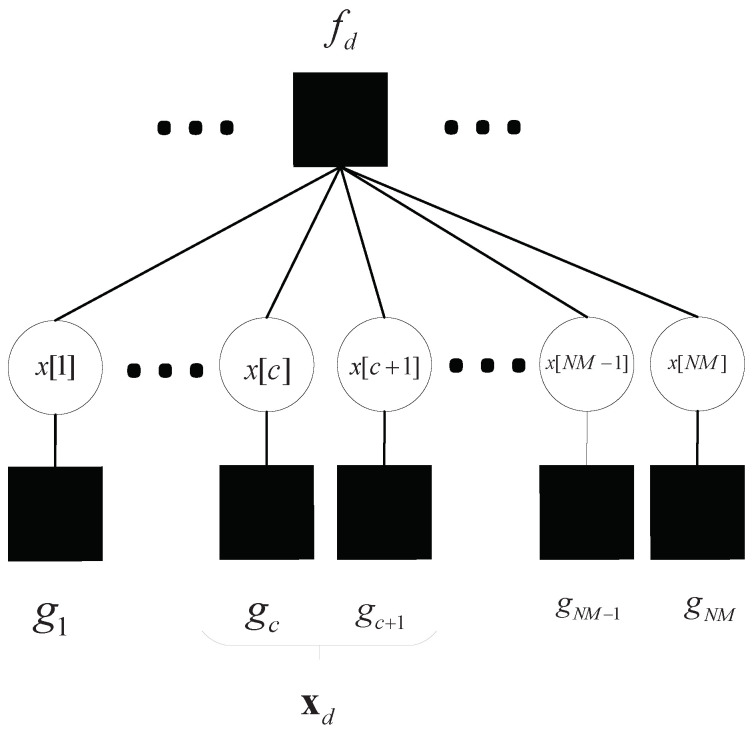
Factor graph depiction of the analyzed OTFS system.

The representation encapsulated in Equation (31) can be interpreted via a factor graph, as illustrated in Figure 3. This schematic elucidation provides a sophisticated methodology for ascertaining the marginal distribution, capitalizing on the fact that the variables are independent conditionally. Within this graph, each rectangular node, termed a function node (FN), denotes a specific function, such as $f_{d}=p\left(y_{d}|x_{d}\right)$, where $x_{d}=\left\{x\left[e\right]|e\in R_{d}\right\}$. On the flip side, every circular node, emblematic of a variable node (VN), aligns with a unique variable. Given the inherent sparsity of the EC matrix H, the interconnections between variables and FCs remain notably finite.

Owing to the impact of the fractional Doppler, it is discernible that the quantity of VNs linked to each FN significantly surpasses the available path count. As a result, the detection procedure for fractional Doppler systems attains a notably elevated level of complexity.

**Figure 4:**
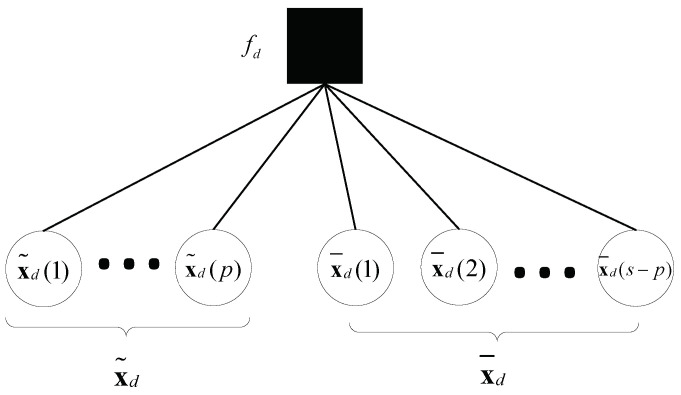
The connections between function and VNs within the local context.

We introduce the concept of $I_{d}^{i}$, which represents the non-zero elements in $R_{d}$ that are related to $h_{i}$. Similarly, we define $x_{d}^{i}$ as the VNs from $x_{d}$ that are associated with $h_{i}$. There are $2N_{T_{i}}+1$ elements for both $I_{d}^{i}$ and $x_{d}^{i}$. To mitigate the complexity of OTFS symbol detection, we select the element whose absolute value is the highest from $I_{d}^{i}$ as the main path, as it affects symbol detection most. The main paths within $R_{d}$ are denoted as a set $\tilde{I_{d}}$, and the remaining elements of $R_{d}$ as $\bar{I_{d}}$. The non-approximate MP algorithm is employed for $\tilde{I_{d}}$, while a GA-based MP algorithm is used for $\bar{I_{d}}$ in the context of symbol detection. Consequently, the collections of VNs are referred to as ${\tilde{x}}_{d}$ and ${\bar{x}}_{d}$, respectively. The partial interconnections between the VNs and FNs are delineated in Figure 4.

The detection algorithm is utilized to realize the factor graph model for calculating the marginal distribution. We begin by considering $\psi_{e/d}\left(x\right)=\mu_{x\to f_{e/d}}\left(x\right)$ and $\phi_{e/d}\left(x\right)=\mu_{f_{d}\to x}\left(x\right)$ as the iteration’s value from node x[c] to node $f_{d}$ and from node $f_{d}$ to node x[c], respectively. Here, define ${\tilde{\phi }}_{e/d}\left(x\right)={\tilde{\mu }}_{f_{d}\to x\left[c\right]}\left(x[c]\right)$ and ${\tilde{\psi }}_{e/d}\left(x\right)={\tilde{\mu }}_{x\left[c\right]\to f_{d}}\left(x[c]\right)$ as the previous iteration’s value of $\mu_{f_{d}\to x\left[c\right]}\left(x[c]\right)$ and $\mu_{x\left[c\right]\to f_{d}}\left(x[c]\right)$, respectively. Specifically, the message transmitted towards a VN $x_{c}$ from an FN $f_{d}$ and the message passing procedure from x[c], a VN, to $f_{d}$, an associated factor node, can be established as follows:

(32) $$\begin{array}{c} \left\{\begin{array}{c} \phi_{e/d}\left(x\left[c\right]\right)=\sum_{x_{d}∼x\left[c\right]}p\left(y\left[d\right]|x_{d}\right)\prod_{e\in R_{d}∼c}\psi_{e/d}\left(x\left[e\right]\right).\\ \psi_{e/d}\left(x\left[c\right]\right)=p\left(x[c]\right)\prod_{e\in C_{c}∼d}\phi_{e/d}\left(x\left[c\right]\right).\; \end{array}\right. \end{array}$$

In order to enhance the performance, we incorporate a “damping factor”, denoted as Δ, where Δ falls within the range of (0,1], during the message update process $\mu_{f_{d}\to x\left[c\right]}\left(x[c]\right)$. Consequently, Equation (32) can be reformulated as:

(33) $$\begin{array}{cc} \phi (x[c])= & \sum_{x_{d}∼x\left[c\right]}p\left(y\left[d\right]|x_{d}\right)\prod_{e\in R_{d}∼c}\psi \left(x\left[e\right]\right)\\ \; & +\Delta \cdot \tilde{\phi }\left(x\right).\; \end{array}$$

We can see that the damping factor has obvious effects on the detection performance in the simulation outcomes.

Likewise, the message passing procedure from x[c], a VN, to $f_{d}$, an associated factor node, is established as follows:

Being estimated through an approximate distribution, $b\left(x[c]\right)$, which is referred to as the “belief”, x[c] can be reformulated as:

(34) $$\begin{array}{c} b\left(x[c]\right)=p\left(x[c]\right)\prod_{e\in C_{c}}\phi_{e/d}\left(x\left[c\right]\right).\; \end{array}$$

In line with the hybrid MP algorithm, $\mu_{x\left[e\right]\to f_{d}}\left(x[e]\right)$ is divided into two distinct components, as described by:

(35) $$\begin{array}{cc} \psi_{e/d}\left(x\left[e\right]\right) & =\sum_{i=1}^{Q}\mu_{e,d}^{\left[i\right]}\delta \left(x\left[e\right]-\alpha_{i}\right),e\in \tilde{I_{d}};\\ \psi_{e/d}\left(x\left[e\right]\right) & =\psi_{e/d}^{G}\left(x\left[e\right]\right),e\in \bar{I_{d}}, \end{array}$$

(36) $$\begin{array}{cc} m_{x[e]} & =E_{p_{x[e]}}\left[x[e]\right]=\sum_{i=1}^{Q}\mu_{e,d}^{\left[i\right]}\alpha_{i}\\ v_{x[e]} & =E_{p_{x[e]}}\left[x{\left[e\right]}^{2}\right]-{\left|m_{x[e]}\right|}^{2}\\ \; & =\sum_{i=1}^{Q}\mu_{e,d}^{\left[i\right]}{\left|\alpha_{i}\right|}^{2}-{\left|m_{x[e]}\right|}^{2}, \end{array}$$

where $\mu_{e,d}^{\left[i\right]}$ represents the probability and $\mu_{x\left[e\right]\to f_{d}}^{G}\left(x[e]\right)$ signifies a function. Upon inserting the expression from Equation (35) into Equation (32), we derive Equation (37), which is displayed at the beginning of the following page.

(37) $$\begin{array}{cc} \; & \phi (x[c])\\ \; & =\int \sum_{x_{d}∼x\left[c\right]}\frac{1}{\pi N_{T_{0}}}\exp\left(\frac{{\left|a\right|}^{2}}{N_{T_{0}}}\right)\\ \; & \prod_{e\in \tilde{I_{d}}∼c}\mu_{e,d}^{\left[i\right]}\delta \left(x\left[e\right]-\alpha_{i}\right)\\ \; & \times \prod_{e\in \bar{I_{d}}∼c}\exp\left(-\frac{{\left|x\left[e\right]-m_{x[e]}\right|}^{2}}{v_{x[e]}}\right)dx_{d}∼x\left[c\right]+\Delta \cdot \tilde{\phi }\left(x\left[c\right]\right)\\ \; & =\sum_{i=1}^{Q}\cdots \sum_{i=1}^{Q}\frac{1}{\pi N_{T_{0}}}\exp\left(\frac{{\left|a-\sum_{e\in \bar{I}\left(d\right)∼c}H\left[d,e\right]m_{x[e]}\right|}^{2}}{N_{T_{0}}+\sum_{e\in \bar{I}\left(d\right)∼c}{\left|H[d,e]\right|}^{2}v_{x[e]}}\right)\\ \; & \times \prod_{e\in \tilde{I_{d}}∼c}\mu_{e,d}^{\left[i\right]}\delta \left(x\left[e\right]-\alpha_{i}\right)+\Delta \cdot \tilde{\phi }\left(x\left[c\right]\right). \end{array}$$

Following this, we employ the message $\mu_{f_{d}\to x\left[c\right]}\left(x[c]\right)$ to adapt the message $\mu_{x\left[c\right]\to f_{d}}\left(x[c]\right)$. $a=y\left[d\right]-\sum_{e\in \tilde{I_{d}}∼c}H\left[d,e\right]x\left[e\right]-H\left[d,c\right]x\left[c\right]$. Consequently, we establish the belief $b\left(x[c]\right)$. The ISl x[c] can be detected by applying the a posteriori rule, i.e.,

(38) $$\begin{array}{c} \hat{x}\left[c\right]=\arg\max_{x[c]\in A}b\left(x[c]\right). \end{array}$$

From Equation (37), it is evident that there are *Q* states in the detection procedure of each data symbol, and the summation needs to encompass all symbols within $x_{d}$ except x[c]. Consequently, the summation operations requires ${\left|Q\right|}^{P}$ operations. Considering that the operational term is insignificantly small in comparison to the exponentially growing term ${\left|Q\right|}^{P}$, the complexity is effectively $O\left({\left|Q\right|}^{P}\right)$. It is worth noting that the computation complexity would be an overwhelming $O\left({\left|Q\right|}^{P(2N_{T_{i}}+1)}\right)$ without the Gaussian approximation, rendering it nearly impossible for implementation.

Hybrid detection schemes may involve more complex algorithms and processing steps compared to simpler, single-method detection schemes. Integrating multiple detection methods introduces challenges in terms of system implementation. The coordination and synchronization of different algorithms, especially in real-time applications, can contribute to increased an design and implementation complexity. The primary motivation for adopting a hybrid detection scheme is often to enhance robustness. By combining multiple methods, the system becomes more adaptable to diverse operating conditions, variations in the signal environment, and potential challenges like noise, interference, or fading. Hybrid schemes can lead to an improved detection accuracy compared to individual methods. Each detection method may excel in specific scenarios or under certain conditions, and their combination can provide a more comprehensive and reliable detection capability.

## 5. Results

In this section, we use Monte Carlo simulations to conduct in-depth research on the evaluation of target estimation and symbol detection performance in different scenarios. We conduct these simulations with an average over $10^{4}$ OTFS frames. For each OTFS frame, we configure the parameters with $M_{S}=32$ and $N_{T}=16$, indicating the presence of 32 sub-carriers and 16 time slots within the TF domain. The ISs are four-QAM modulated from the data bits generated randomly. In addition, simulation results show that the proposed hybrid MP detector has a high effectiveness. The effectiveness of the hybrid message-passing algorithm is substantiated, and comprehensive comparisons are made, specifically in relation to conventional detection algorithms encompassing Gaussian-approximation-based MP methods. A uniform maximum iteration limit, denoted as $I_{\max}$, is uniformly established as 10 for all algorithms under consideration.

**Figure 5:**
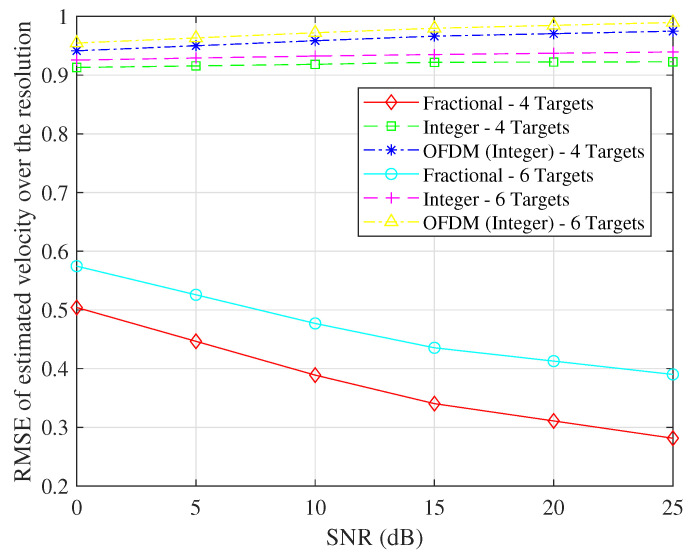
The RMSE in velocity estimation concerning the SNR.

**Figure 6:**
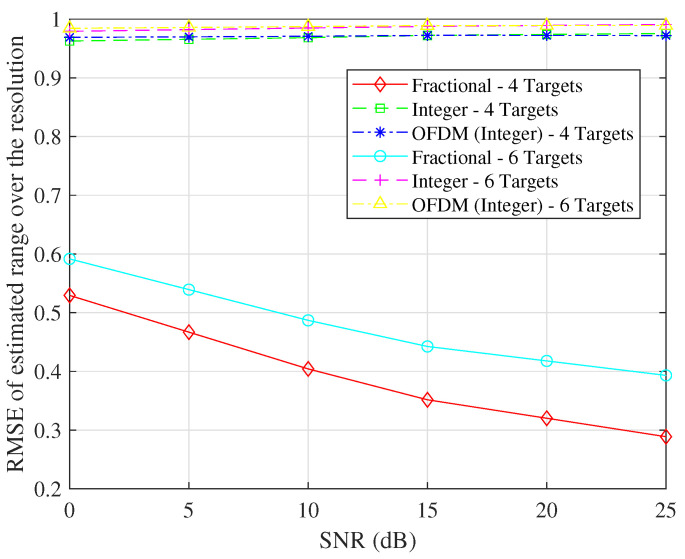
The RMSE in range estimation concerning the SNR.

We investigate two different simulations, one involving four targets and the other involving six targets. Our analysis involves the computation of the ratio between the root mean square error (RMSE) and the corresponding resolution in estimating both R and V. We vary the SNR and determine the RMSEs in velocity estimation and range estimation concerning the SNR. The results of these simulations are visually presented in Figure 5 and Figure 6.

As depicted in Figure 5, a noticeable trend emerges when there are four targets: the RMSE associated with the estimated velocity significantly diminishes when the fractional Doppler index is determined through (26). This observation underscores the efficacy of our proposed algorithm. Conversely, when the scenario involves six targets, the RMSE performance deteriorates at identical SNR levels. This degradation is attributed to the heightened interference arising from the overlapping responses of each symbol, rendering the approximation in (25) less precise. Consequently, the accuracy of velocity estimation suffers.

Similarly, as illustrated in Figure 6, when the simulation entails four targets, the RMSE in range estimation is reduced, and the results of the target estimation substantially deteriorate only if the integer part of the indices is considered. This phenomenon aligns with the increased interference and inaccuracies associated with the estimation process in scenarios with a greater number of targets.

When considering only the integer components, it becomes evident that the RMSE in range estimation remains largely unaffected with respect to varying SNRs. This phenomenon arises due to the limited resolutions of R and V within each grid. The average minimum errors align with half of the resolution values, a correspondence that is consistent with our simulation findings.

In the simulations of the proposed hybrid MP detector, we employ a random selection of DD parameters for each path, ensuring the fractional Doppler indices. Note that, for both the Gaussian-approximation-based MP and hybrid MP algorithms, the damping factor is set to 0.7.

**Figure 7:**
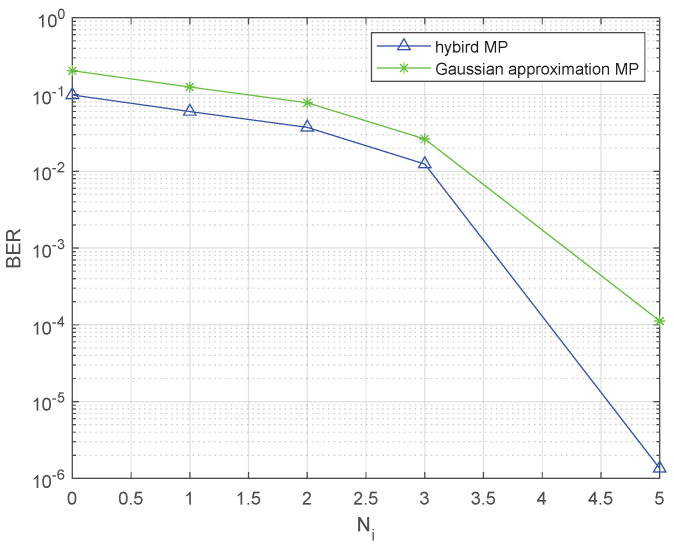
The BER performance concerning various values of $N_{T_{i}}$.

We initiate our analysis by examining the influence of the parameter $N_{i}$. Specifically, we apply both the Gaussian-approximation-based MP and the hybrid MP algorithms to various values of $N_{T_{i}}$ under specific SNR conditions, where the SNR=18dB and P=6. The acquired findings are depicted graphically in Figure 7.

Remarkably, a substantial improvement in the bit error rate (BER) is discernible as $N_{T_{i}}$ increases. As $N_{T_{i}}$ reaches 2 and beyond, it becomes apparent that the performance of the Gaussian-approximation-based MP stabilizes, while the hybrid MP’s performance continues to improve. Furthermore, it is worth noting that, across all values of $N_{i}$, the hybrid MP consistently outperforms the Gaussian-approximation-based MP.

**Figure 8:**
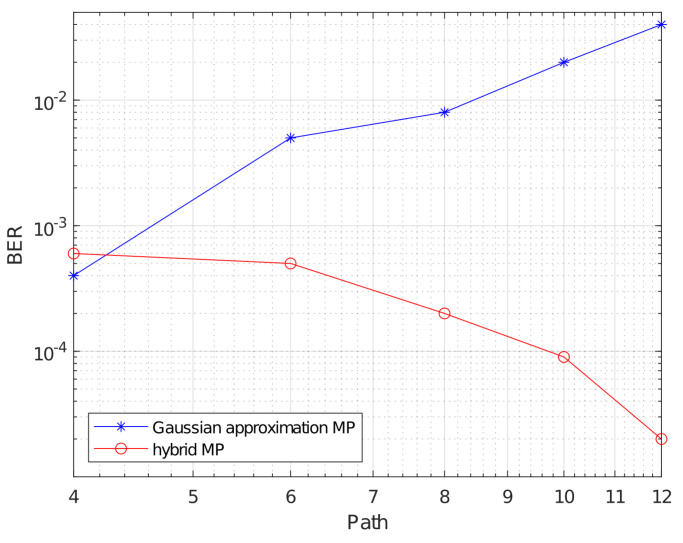
The BER performance under different values of *P*.

Further, we shift our focus to the BER performance comparison of the Gaussian-approximation-based MP algorithm and the hybrid MP with different values of *P*. The outcomes of this analysis are illustrated in Figure 8, maintaining an SNR=18dB and fixing $N_{T_{i}}=2$.

It is evident from the results that the hybrid MP consistently outperforms the GA-based MP when the system involves more than four paths. For scenarios with precisely four paths, both the Gaussian-approximation-based MP and the hybrid MP exhibit similar performances, characterized by a BER of approximately $5\times 10^{-4}$. However, when there are more paths, the performance of the Gaussian-approximation-based MP deteriorates, while the hybrid MP’s performance remains robust. This observation underscores the superior effectiveness of the hybrid MP in mitigating multipath interference.

The underlying reason for this disparity lies in the convergence behavior of the two algorithms. The Gaussian-approximation-based MP tends to converge towards a local optimum due to the presence of a substantial number of loops. In contrast, the hybrid MP algorithm efficiently leverages diversity gain, leading to its superior performance in such scenarios.

**Figure 9:**
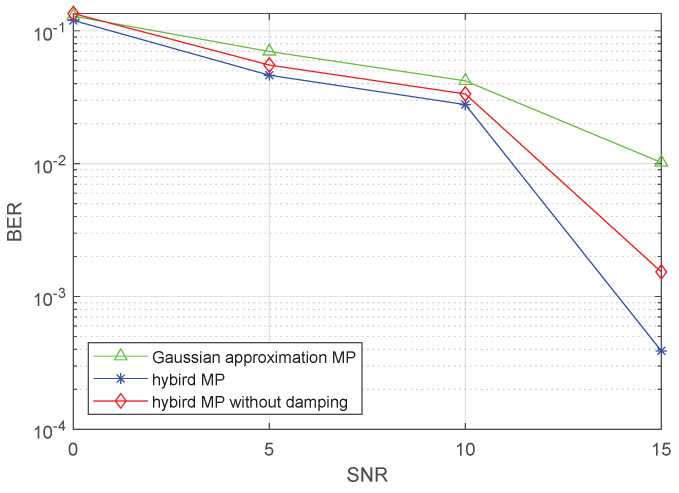
BER performance.

In Figure 9, we present the BER performance of the hybrid MP algorithm across various SNRs with the same P=8 and $N_{T_{i}}=2$. We compare this performance with that of the MMSE detector, the GA-based MP, and the hybrid MP without any damping factor.

The results indicate that the hybrid MP exhibits robust performance across the entire SNR range. Particularly noteworthy is the performance of the hybrid MP without the “damping factor”, which closely matches that of the Gaussian-approximation-based MP. The inclusion of the “damping factor” significantly improves the performance, reducing the BER from $5\times 10^{-4}$ to $2\times 10^{-5}$. This enhancement is especially notable.

It is worth mentioning that a previous study suggested that a “damping factor” might not be necessary for the hybrid algorithm. However, the presence of the fractional Doppler leads to interference energy being spread across more paths, necessitating the incorporation of a “damping factor” to enhance effectiveness.

## 6. Discussion

The adaptability of the OTFS framework to future technological advancements and standards depends on its inherent flexibility, the willingness of the research community to evolve the framework, and its ability to integrate with emerging technologies. The ability of the OTFS framework to integrate with emerging wireless communication standards is crucial for its long-term adaptability. Compatibility with new modulation schemes, protocols, and frequency bands enhances its relevance in evolving wireless networks. The OTFS framework is designed to support multiple access schemes. It can be more adaptable to diverse communication scenarios. The ability to accommodate different access methods allows it to align with future network architectures and requirements. As wireless networks evolve, the scalability and performance optimization of the OTFS framework will become increasingly important. Continuous efforts to enhance scalability and optimize performance ensure that the framework remains viable in large-scale and high-performance communication systems. The ability of the OTFS framework to address emerging use cases, such as Internet of Things (IoT), Industry 4.0, and beyond, is a key factor in its adaptability. Anticipating and accommodating diverse communication scenarios ensures continued relevance.

There are some limitations to our method. The scalability of the OTFS framework in handling a large number of simultaneous targets or users depends on various factors. Efficient parallelization of algorithms can enhance the system’s ability to handle a large number of simultaneous targets or users. The OTFS framework can potentially support multiple access schemes, allowing it to handle multiple targets or users simultaneously. Differentiation and isolation of signals in the DD domain contribute to scalability. The ability of OTFS modulation to adapt to dynamic channel conditions is beneficial for scalability. It enables the framework to handle variations in the number of targets or users and changes in the communication environment. The computational complexity of the OTFS framework may become a limiting factor as the number of simultaneous targets or users increases. Processing overheads may scale with square or higher orders of the number of targets, impacting real-time performance. Accurate channel estimation and tracking become more challenging with a larger number of simultaneous targets or users. The complexity of tracking and adapting to dynamic channels may impact the scalability of the system. In scenarios with a high density of simultaneous targets or users, interference and crosstalk between signals may increase. The ability of OTFS modulation to distinguish between closely spaced targets may be limited, affecting system performance.

The OTFS framework is designed to be robust in the face of challenging channel conditions in rapidly changing multipath environments. Its unique approach to signal representation in the DD domain offers advantages in scenarios where conventional modulation schemes may struggle. The OTFS framework excels in representing signals in the DD domain, providing a more natural and robust representation of the channel characteristics. This representation allows for the separation of signals arriving at different delays and Doppler shifts, even in environments with severe multipath conditions. OTFS modulation can mitigate the effects of frequency-selective fading, a common challenge in multi-path environments. By transforming signals to the DD domain, OTFS modulation provides a way to disentangle frequency-selective fading components, leading to an improved communication performance. The OTFS framework can be designed to incorporate dynamic channel tracking algorithms. These algorithms adapt to changes in the channel conditions, continuously updating channel estimates to maintain a robust communication performance in environments with varying multi-path characteristics.

Accurate channel estimation is crucial for obtaining channel state information (CSI) for reliable detection. In the OTFS, the instantaneous delay shift and Doppler shift of channel information are discrete on the DD grid. The time delay resolution and Doppler resolution depend on the bandwidth and duration, respectively. In actual communication scenarios, the bandwidth is large enough to provide sufficient latency resolution, but due to the low latency requirements of future communication, the duration may be relatively small. Therefore, the fractional Doppler needs to be considered. In the case of the integer Doppler, the effective channels in the DD domain are sparse. On the contrary, for the fractional Doppler case, the effective DD main channel is distributed across all Doppler indices, which reduces the sparsity of the effective channel and may therefore reduce the channel estimation performance. Estimating the fractional Doppler enables us to improve channel estimation by reconstructing effective DD domain channels using estimated Doppler frequency shifts, which is essential for reliable detection. Furthermore, this can improve the accuracy of positioning. When OTFS modulation assists sensing, accurate estimation of mobile user speed can be obtained through accurate estimations of Doppler frequency shifts.

## 7. Conclusions

In this paper, we proposed a new method to estimate the fractional DD indices of off-grid targets using a 2D correction-based approach. The DD indices of off-grid targets appear as fractional values in the DD domain. To address this, we presented a novel method based on differences to estimate the fractional components of these parameters. According to the experimental results, the proposed algorithm can accurately estimate the R and V of off-grid targets. In addition, we also designed a hybrid MP-based detector for communication, which has a lower BER performance than existing detection methods according to the simulation results.

By integrating the OTFS framework with existing communication and sensing systems, adaptation of signal processing modules can be achieved. The OTFS framework relies on a unique signal processing approach. Adaptation of existing signal processing modules is necessary to incorporate the transformations necessary for OTFS modulation. OTFS modulation may have specific requirements for error correction in the DD domain and adapt decoding algorithms to handle signals transformed into the DD domain. The OTFS framework can be used thorough simulations and testing to evaluate the performance of an integrated system, and based on performance evaluations, it can iterate through optimization cycles to fine-tune parameters, algorithms, and configurations for an optimal performance. Meanwhile, it can evaluate the latency in the context of real-time requirements and optimize processing pipelines to meet the latency constraints of the overall system. 

## Data Availability

Data are contained within the article.
